# Physical activity promotion in chiropractic: a systematic review of clinician-based surveys

**DOI:** 10.1186/s12998-022-00467-9

**Published:** 2022-12-13

**Authors:** Matthew Fernandez, Anika Young, Karen Milton, Marina Pinhiero, Katie de Luca, Paulo Ferreira, Jeffrey Hebert

**Affiliations:** 1 School of Health, Medical and Applied Sciences, Central Queensland University, Brisbane, Australia; 2 Department of Chiropractic, Faculty of Medicine, Health and Human Sciences, Macquarie University, Sydney, Australia; 3 Norwich Medical School, University of East Anglia, Norwich, UK; 4 Institute for Musculoskeletal Health, The University of Sydney and Sydney Local Health District, Sydney, Australia; 5 School of Public Health, Faculty of Medicine and Health, The University of Sydney, Sydney, Australia; 6 Sydney Musculoskeletal Health, School of Health Sciences, Charles Perkins Centre, Faculty of Medicine and Health, University of Sydney, Sydney, NSW Australia; 7 Faculty of Kinesiology, University of New Brunswick, Fredericton, Canada

**Keywords:** Physical activity, Exercise, Fitness, Promotion, Counselling, Advice, Practice, Systematic review, Healthcare, Chiropractic

## Abstract

**Background:**

Physical inactivity is a global health pandemic. Allied healthcare providers, including chiropractors, are well placed to integrate individual physical activity (PA) promotion into routine care. A previous systematic review identified that approximately 90% of chiropractors held a positive opinion towards healthier patient lifestyles; however, the extent to which chiropractors promote PA to their patients within routine care is unclear. This systematic review aimed to describe chiropractors' attitudes towards and current practice in advising, counselling, discussing, supporting, or recommending PA to patients.

**Methods:**

Five databases were searched from inception to December 2021 for cross-sectional surveys that explored PA promotion by chiropractors in practice. We assessed the risk of bias of the included studies with the ‘Risk of Bias in Cross-Sectional Surveys of Attitudes and Practices’ tool. Descriptive data were extracted, grouping similar survey questions and responses into emerging categories. Chiropractors’ views regarding the perceived importance and/or their preparedness to counsel and provide PA or exercise information are reported.

**Results:**

From 661 studies, 15 met the selection criteria. Surveys included 7999 chiropractors primarily from the USA, UK, Australia, and Sweden. All studies were rated as moderate-to-high risk of bias, with methodological weaknesses characterised by inconsistent reporting of missing data, non-representative samples, low response rates (i.e., less than 60%), and unknown validity of survey instruments. Chiropractors frequently recognised the importance of PA promotion, as demonstrated by the proportion of respondents reporting that they: (1) support the importance of providing PA or exercise information and counselling (64% to 100%); (2) are prepared to provide PA or exercise information and/or counselling to patients (91% to 92%,); (3) frequently obtain PA or exercise information from patients (87% to 97%,); 4) frequently discuss PA or exercise and/or provide PA or exercise information to patients (68% to 99%); and 5) frequently provide PA counselling to patients (50% to 81%.).

**Conclusion:**

A large majority of practising chiropractors actively engage with PA promotion. However, the results should be interpreted with caution owing to the moderate-to-high risk of bias of the included studies. Forthcoming research initiatives should explore unbiased surveys, further PA education and training as well as capitalising on chiropractors’ own PA participation.

**Supplementary Information:**

The online version contains supplementary material available at 10.1186/s12998-022-00467-9.

## Background

Physical activity (PA), including structured exercise, is widely recognised as an important behaviour for reducing the risk of all-cause mortality, and chronic diseases, including (but not limited to) cardiovascular diseases, type 2 diabetes and various forms of cancer [1]. Further, recognised health benefits associated with PA include reduced risk of depression and anxiety, falls and fall-related injuries, and improved cognitive function and sleep [1]. Yet, physical inactivity continues to be a major worldwide pandemic [2], with little-to-no improvement in overall global PA participation observed over the last two decades [3, 4]. Appropriately, the recent 2020 World Health Organization (WHO) guidelines on PA and sedentary behaviour emphasise that all adults should undertake 150–300 min of moderate intensity, or 75–150 min of vigorous-intensity PA (or a combination of moderate and vigorous PA) per week, in addition to resistance (strength) training [5]. For those not meeting these recommendations, some PA is considered better than none, with small amounts recommended to gradually increase PA for optimal health outcomes.

In light of recent global estimates showing that one in four (28%) adults are not meeting PA recommendations [3], the WHO’s Global Action Plan on PA 2018–2030 provides both guidance and a framework to counteract global physical inactivity, aiming for a 15% reduction by 2030 [6]. To increase PA participation worldwide, a ‘whole of systems approach’ has been advocated [7, 8], to enhance social, cultural, economic and environmental factors—which ultimately influence individual PA approaches [6]. Briefly, the strategic objective of the WHO systems approach is to scale-up policy actions through the creation of active societies, active environments, active people, and active systems [6], thereby expanding the multifaceted reach of PA. The healthcare sector is well placed within the Global Action Plan to incorporate individual PA counselling (or alike) into routine treatment and care [9]. Notably, a recent systematic review of randomised controlled trials identified that PA interventions, delivered in primary care settings by health professionals, are effective in increasing PA participation [10].

Allied health care providers (AHPs) such as chiropractors, may be well positioned to deliver PA promotion within the healthcare sector. Worldwide, the median 12-month utilisation of chiropractic services has been estimated to be approximately 9%, primarily for musculoskeletal conditions [11]. Yet, the role that chiropractors play in influencing lifestyle-related risk factors, like PA, remains under researched and underutilised [12, 13]. A systematic review on primary prevention in chiropractic practice showed that approximately 90% of chiropractors had a positive opinion towards healthy lifestyle promotion and were interested in their patients engaging in healthier lifestyles [14]. However, this review was limited to 3 studies and excluded grey literature, thus it may not represent a comprehensive synthesis and appraisal of existing evidence. Furthermore, it did not attempt to assess the extent to which chiropractors promote PA to their patients within routine care.

While the chiropractic literature regarding PA promotion is relatively sparse, among other AHPs such as physiotherapists, PA data exist to both inform clinical practice and highlight where evidence-based practices are not being implemented [15]. For instance, while Australian physiotherapists believe it is their role to counsel patients on PA, evidence suggests they have poor knowledge of the PA and sedentary behaviour guidelines [16, 17]. More recently, results from a multi-national survey recommended professional development initiatives for physiotherapists to adequately deliver PA advice and exercise prescription [18]. To support the integration of PA promotion into routine chiropractic practice, it is critical to understand perceptions and current practices.

We present a systematic review of available cross-sectional surveys of chiropractic clinicians on PA promotion. Specifically, we aimed to describe chiropractors' attitudes towards and current practice in advising, counselling, discussing, supporting, or recommending PA to patients. This knowledge will inform further research and opportunities to strengthen future practice delivery.

## Methods

### Design

A systematic review of published literature to establish and describe current chiropractic practice in PA promotion. This systematic review was pre-registered with PROSPERO (CRD42022297430) and conducted in accordance with the Preferred Reporting Items for Systematic reviews and Meta-Analyses (PRISMA) 2020 statement [19].

### Search criteria

We developed a structured search strategy comprising text and MeSH terms (Additional file 1: Table 1). We searched five databases from inception to August 2020: Medline, Mantis, AMED, EMBASE, and Index to Chiropractic literature. The same search strategy was rerun in December 2021. In addition, the reference lists of included articles were checked manually for publications of relevance. Forward and backward citation tracking was conducted up to December 2021 to identify other relevant studies. A hand search of relevant websites for unpublished surveys was also performed. No restriction was applied to language.

### Eligibility criteria

The study was designed according to the following PICOS strategy [20] and searches were screened according to the following eligibility criteria:

I. Population: chiropractic clinicians who are currently licenced/registered and currently in clinical practice.
II. Intervention: licenced/registered chiropractor clinicians incorporating of any form of PA advice, counsel, discussion, support and recommendation or exercise prescription, within a clinical practice setting.
III. Comparator: not applicable.
IV. Outcome: at least one measure that assesses self-reported attitudes towards PA promotion among chiropractic clinicians in clinical practice.
V. Study design: cross-sectional studies, i.e., surveys or questionnaires.

Studies were ineligible if they:

1. were non‐cross‐sectional studies; or
2. reported qualitatively results only; or
3. were reviews, editorials, non-research letters

## Study eligibility, quality assessment and data extraction

### Study eligibility

Two reviewers (MF and AY) independently screened titles and abstracts for eligibility. Studies considered potentially eligible by at least one reviewer were obtained in full text. All potentially relevant full text studies were checked against the selection criteria by two independent reviewers (MF and AY). Disagreements were resolved by discussion, engaging a third reviewer if needed (JH).

### Quality assessment

The quality assessment of each study was performed by two independent reviewers (MF and AY) using the ‘Risk of Bias in Cross-Sectional Surveys of Attitudes and Practices’ tool [21]. Risk of bias was reported on a domain-by-domain basis and then assigned an overall risk of bias rating. The following five domains were appraised: (1) representativeness of the sample, to provide an unbiased estimate of the practices of the population studied. This criterion was met where the target survey population was adequately specified, i.e., the number of eligible chiropractors invited to complete the survey is reported, such as a representative population from a national association database; (2) adequacy of the response rate to reduce any influence on results due to differences between respondents and non-respondents. This criterion was met where a participation rate of at least 60% or above was achieved; (3) missing data within completed questionnaires, i.e., whether bias may have been introduced if items were not answered by survey respondents. This criterion was met when there was less than 10% missing data when considering all questionnaire items; (4) conduct of pilot testing. This criterion was met if a formal assessment of the comprehensiveness, clarity and face validity of a questionnaire was carried out prior within a subset of a similar population of individuals; and (5) validity of the survey instrument, i.e., survey items evaluated the theoretical concept(s) that the survey intended to measure. This criterion was met if there was evidence of established reliability and construct validity or modeled on prior questionnaires and produced responses similar to other established surveys for a similar population. All domains were classified as “low risk of bias” or “high risk of bias”. For each domain, response options were rated as "low risk of bias" for ‘probably yes’ and ‘definitely yes’, and "high risk of bias" for ‘probably no’ and ‘definitely no’. The description of ‘probably yes’ or ‘probably no’ was applied to studies that did not provide conclusive information for definitive “yes” or “no” judgments, however it was very likely (or unlikely) that the study met that particular criterion. The description of ‘definitely yes’ or ‘definitely no’, was applied when each item provided a definitive “yes” or “no”. A global rating was then determined based on the scores of each domain. Studies scoring low risk on at least four domains were classified as low risk overall, studies scoring high risk on at least four domains were classified as high risk overall and studies with more mixed findings across the five domains were classified as moderate overall risk.

### Data extraction

Relevant data were extracted from the included studies (i.e., country of the survey, population, sampling, definitions, response rate, outcomes) by two independent reviewers (MF and AY) using a standardised form. The main results of each study (i.e., prevalence point estimates) were also extracted. Disagreements were resolved by discussion with a third reviewer if needed (JH).

## Data analysis/synthesis

Two independent reviewers (MF and AY) independently synthesised descriptive and analytic data by grouping similar survey questions and subsequent responses into emerging categories. These categories were developed to closely resemble the original material based on the common PA questions asked within the different surveys. To understand current PA promotion practices in chiropractic, we extracted data based on the percentage of chiropractors who answered relevant survey questions via Likert scale response options. Only the percentage of participants who responded favourably to the provided statement or question on PA were extracted. For example, for the statement ‘chiropractic care should include PA recommendations’, favourable responses included ‘strongly agree’ or ‘agree’. Similar responses were categorised and subsequently organised into tables reflecting five main themes: (1) the importance of counselling and providing PA or exercise information; (2) readiness to counsel and/or provide PA or exercise information; (3) obtaining PA or exercise information from patients; (4) discussing PA or exercise and/or providing PA or exercise information; and (5) counselling. Considering the descriptive and analytical nature of the included studies, we performed a narrative synthesis of the results. We provide the prevalence point estimates for each included study in a tabular format (using the Wilson score method) and the proportion of respondents from the arising categories in the text. Any disagreements between authors were resolved through discussion with a third reviewer if needed (JH).

## Results

### Study characteristics

Our search strategy identified 661 studies, 15 of which met the inclusion criteria, representing a collective sample of 7999 registered chiropractic clinicians (Additional file 2: Fig. 1). The reasons for study exclusion are reported in Additional file 3: Table 2. Study sample sizes ranged from 38 to 1924 participants, studies were published between 1990 and 2021, and response rates ranged from 21 to 65%. Eleven studies were from the USA [22–32], two were from Australia [33, 34] and one each from the UK [35] and Sweden [36]. Of the studies in which participants’ gender were reported (n = 14), the proportion of male chiropractors was considerably higher than females (approximately 75% males). Included studies were heterogeneous in their survey methods. Seven studies used a postal mail-survey [22, 23, 25, 26, 30, 31, 34], four were electronically delivered [27, 29, 35, 36], one used electronic and postal mail [33], and one used a combination of electronic and face-to-face distribution during a convention [24]. Two studies did not specify postal or electronic methods [28, 32]. Participants were primarily recruited either randomly from specific chiropractic directories [25, 26, 30, 31, 34] or surveys were distributed via chiropractic professional bodies or associations to respective members [22–24, 28, 29, 32, 33, 35, 36]. Survey instruments consisted primarily of closed questions with Likert scale response options. The characteristics of all included studies are summarised in Table 1.

**Table 1:** Characteristics of cross-sectional studies estimating the self-reported prevalence of physical activity promotion in clinical practice among chiropractors

Author (year)	Country	Source population	Sampling approach	Response rate %	Sample size respondents from the total source population	Assessment method	Age	Gender (% male)	Overall risk of bias
Adams (2017)	Australia	Registered chiropractors throughout Australia	Convenience Sampling	43	2005 out of 4684	Electronic and postal mail	Mean of the sample was 42 years (SD = 12.1)	62	High
Boline (1990)	USA	Licensed chiropractors in the State of Iowa were surveyed	Convenience Sampling	51	374 out of 738	Postal mail	Not specifically reported, other than more than half of the sample were younger than 40 years. Mean and SD not reported	88	High
Fikar (2015)	UK	Chiropractors who were members of four participating UK chiropractic associations were surveyed	Convenience Sampling	21	509 out of 2448	Electronically	Not reported	55	High
Hawk (1995)	USA	USA chiropractors listed in the National Directory of Chiropractic 1993–94 edition	Random sample	65.3	492 out of 753	Postal mail	25–34 years: 18%; 35–44 years: 22%; 45–54 years: 48%; 55–64 years: 17%; over 65 years: 8%. Mean and SD not reported	81	Moderate
Hawk (2001)	USA	Chiropractors were licensed, in practice and part of a practice-based research program	Convenience Sampling	Not reported	155. Total source population not reported	Electronically and postal mail methods were not specified	Not reported	83	High
Hawk (2004)	USA	Chiropractors, from the state licensing boards list	Random sample	25	496 out of 2000	Postal mail	25–34 years: 22%; 35–44 years: 34%; 45–54 years: 30%; > 55 years: 12%. Mean and SD not reported	80	High
Hawk (2011)	USA	Chiropractic currently in practice, and were members of the Integrated Chiropractic Outcomes Network Nationally	Convenience Sampling	Not reported	38. Total source population not reported	Electronically and postal mail methods were not specified	Not reported	74	High
Himelfarb (2020)	USA	USA practicing chiropractors	Representative sample	24	1975 out of 8242	Electronically	< 30 years: 5%; 30–59 yeas: 65%; over 60 years: 30%. Mean and SD not reported	68	High
Ivie (2011)	USA	Current Alabama State Chiropractic Association members	Convenience Sampling	53	105 out of 197	Electronically	< 30 years: 8%; 30–39 years: 32%; 40–49 years: 26%; > 50 years: 34%. Mean and SD not reported	82	Moderate
Jamison (2002)	Australia	Member directory of the Chiropractic Association of Australia	Random sample	35	138 out of 400	Postal mail	< 25 years: 2%; 25–40 years: 58%; 41–60 years: 32%; > 60 years: 7%. Mean and SD not reported	80	High
Leach M (2021)	Sweden	Licensed chiropractors of the Swedish Chiropractic Association	Convenience Sampling	33	56 out of 172	Electronically	20–29 years: 5%; 30–39 years: 30%; 40–49 years: 29%; 50–59 years: 23%; > 60 years: 13%. Mean and SD not reported	57	Moderate
Leach R (2011)	USA	Mississippi Chiropractic Association members	Convenience Sampling	43	68 out of 157	Surveys were 1) administered and completed by Mississippi chiropractors present at the association meeting and 2) sent electronically and by postal mail to members who were not present at the meeting	22–33 years: 13%; 34–45 years: 37%; 46–57 years: 25%; 58–69 years: 21%; 70–81 years: 3%. Mean and SD not reported	85	High
McDonald (2004)	USA/Canada/Mexico	Dynamic Chiropractic National list of USA, Canadian and Mexican chiropractors	Random sample	63	687 out of 1086	Postal mail	≤ 39 years: 32%; 40–59 years: 61%; ≥ 60 years: 8%. Mean and SD not reported	66	Moderate
Rupert (2000)	USA	National Directory of licensed Chiropractors	Random sample	44	658 out of 1500	Postal mail	25–34 years: 30%; 35–44 years: 46%; 45–54 years: 13%; > 55 years: 11%. Mean of the sample: 44 years (no SD reported)	83	High
Sawyer (1990)	USA	Chiropractors in the State of Iowa were surveyed	Convenience Sampling	50	371 out of 738	Postal mail	≤ 40 years: 54%; 41–55 years: 26%; > 55 years: 20%. Mean of the sample was 43 years (no SD reported)	89	High

(*n*) number, *SD* standard deviation

### Results of risk of bias assessment

Overall, the summary risk of bias was rated as high for 11 studies [22–25, 27, 28, 31–35] and moderate for 4 [26, 29, 30, 36]. The most frequent limitations across studies, on a domain-by-domain basis, was in relation to “Missing data”, with 93% [14 of 15 studies] being classified as high risk of bias. Similarly, 87% (13/15 studies) were classified as high risk of bias in relation to the “Validity of the survey instrument”, “Adequacy of response rate” and “Representativeness of the sample”. For “Pilot testing”, 67% (10/15 studies) were classified as low risk of bias. The risk of bias assessment across all five domains is reported in Table 2 and overall, in Table 1.

**Table 2:**
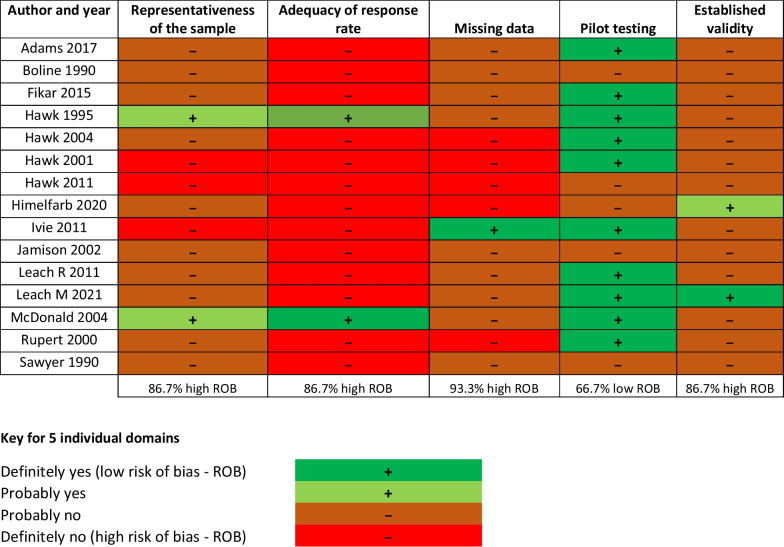
Risk of study bias domain-by-domain assessment

While the underpinnings of included studies were similar, there was variation between the PA variables that were investigated. These variables are presented below and fell broadly into five practice categories, including: (1) the importance of counselling and providing PA or exercise information; (2) readiness to counsel and/or provide PA or exercise information; (3) obtaining PA or exercise information from patients; (4) discussing and/or providing PA or exercise recommendations, including information or resources; and (5) PA counselling. The proportion of reported practice and the factors identified within each theme that influenced PA delivery in chiropractic services are described below and summarised in Table 3.

**Table 3:** Risk of study bias domain-by-domain assessment

Physical activity promotion	First author (year of publication)	Prevalence point estimates (95% confidence intervals)	Favorable response to the survey question asked in relation to physical activity promotion
1. Discussing and providing PA or exercise recommendations for fitness, including information or resources	Adams (2017)	84.9% (83.3–86.4%)	Often discuss PA as part of their care/management plans
	Fikar (2011)	91.6% (88.8–93.7%)	Provide advice/resources given for level of PA performed
	Hawk (1995)	68.1% (63.8–72.1%)	Frequently discussed fitness exercise with patients
	Hawk (2004)	89.0% (85.8–91.4%)	Provide information to all patients in the appropriate age/sex/risk category on exercise for fitness/disease prevention
	Jamison (2002)	99.0% (96.0–99.9%)	Practitioners offer exercise information, including reading material available in the waiting room, group classes, and is not limited to individual consultation counselling
	Leach R (2011)	86.0% (76.7–92.9%)	Incorporate national fitness objectives into their patient recommendations
	Leach M 2021	76.8% (64.2–85.9%)	Provided exercise or PA advice or instruction in the initial chiropractic consultation
	McDonald (2004)	97.8% (96.4–98.7%)	Clinical routine usually includes exercise recommendations
2. Counselling	Hawk (2001)	50.0% (42.5–58.1%)	Exercise counseling was commonly used with more than 50% of patients
	Himelfarb (2020)	80.6% (78.8–82.3%)	Make a specific recommendation to a patient regarding physical fitness and exercise promotion
	Ivie (2011)	78.3% (69.5–85.1%)	Provided PA counseling to ≥ 51% of patients
3. Prepared/readiness to counsel and provide PA, exercise for fitness information or resources	Boline (1990)	91.5% (88.2–93.9%)	Very prepared-or-prepared to counsel patients in health behaviours such as exercise
	Jamison (2002)	91.0% (85.4–95.0%)	Practitioners were prepared to offer exercise counselling
	Sawyer (1990)	91.0% (87.8–93.5%)	Very prepared-or-prepared to provide advice and counselling to patients about exercise habits
4. Importance of counselling and providing PA, exercise for fitness information or resources	Boline (1990)	78.1% (73.6–82.0%)	Very important-or-somewhat important in being involved in health behaviour of engaging in aerobic activity at least 3 times per week
	Fikar (2011)	95.9% (93.8–97.3%)	Believed this lifestyle issue was their responsibility to discuss
	Hawk (1995)	63.9% (58.7–67.2%)	Considered it important for all D.C.'s to discuss fitness exercise with patients
	Hawk (2004)	95.0% (92.7–96.6%)	Should provide information to all patients in the appropriate age/sex/risk category on exercise for fitness/disease prevention
	Ivie (2011)	99% (94.9–99.8%)	It is appropriate to counsel patients regarding vigorous PA
	Jamison (2002)	93.0% (87.2–96.0%)	believed that it is important to include exercise recommendations as part of their care
	Leach R (2011)	94.0% (85.8–97.7%)	Strongly-to-somewhat in agreement with the aim of giving patients advice about PA, exercise
	Rupert (2000)	96.1% (94.3–97.3%)	Strongly agree-to-agree maintenance care should include exercise recommendations
	Sawyer (1990)	78.0% (73.4–81.8%)	Very important-or-somewhat important in being involved in health behaviour of engaging in aerobic activity at least 3 times per week
5. Obtaining information from patients regarding PA or exercise for fitness	Boline (1990)	96.7% (94.3–98.3%)	Routinely-or-occasionally obtained information on behavioural exercise habits information
	Hawk (2004)	87.0% (83.9–89.8%)	Obtain information on PA behaviours to identify at-risk patients
	Fikar (2011)	90.8% (87.9–93.0%)	Evaluated/monitored behaviours related to PA performed
	Hawk (2011)	92.0% (90.8–100.0%)	Routinely-to-frequently obtaining PA information from new patients
	Sawyer (1990)	96.0% (93.4–97.5%)	Routinely-or-occasionally obtained information on behavioural exercise habits

*PA* physical activity, *D.C.'s* Doctor of Chiropractic, *CI* confidence interval

1. Importance of counselling and providing PA or exercise information

Nine studies examined the frequency in which clinicians (n = 3211) considered it important to counsel on PA and provide PA or exercise for fitness information [22–26, 29, 31, 34, 35]. The proportion ranged from 64 to 100% for this category. Six studies reported that more than 90% of clinicians believed it was important to provide PA information, i.e., promoting PA was their responsibility and/or that they should counsel and provide information on PA or include exercise recommendations to their patients [24, 25, 29, 31, 34, 35]. In three studies there was less agreement on the importance of this, ranging from 64 to 78% of clinicians believing it is important for them to discuss fitness and/or exercise and provide information on PA to their patients [22, 23, 26].

2. Readiness to counsel and/or provide PA or exercise information

Three studies examined the frequency in which clinicians (n = 883) felt ready to counsel and/or provide PA or exercise for fitness information [22, 23, 34]. The proportion ranged from 91 to 92% for this category, indicating that clinicians endorsed being prepared to provide PA advice and counselling to patients [22, 23, 34].

3. Obtaining PA or exercise information from patients

Five studies sought to examine the frequency in which clinicians (n = 1788) gather information on PA participation from patients [22, 23, 25, 28, 35]. The proportion ranged from 87 to 97% for this category. Two studies reported that over 95% of clinicians routinely-or-occasionally obtained information on behavioural exercise habits [22, 23]. Other studies showed that over 85% of clinicians frequently-to-routinely obtained information regarding patients’ levels of PA [28], evaluated and monitored behaviours related to the amount of PA performed [35] or obtained information about PA behaviours to identify at-risk patients [25].

4. Discussing and/or providing PA or exercise recommendations, including information or resources

Eight studies sought to examine the frequency in which clinicians (n = 4862) discussed PA or exercise with their patients [26, 33, 36], as well as providing PA, exercise information or recommendations [24, 25, 30, 34, 35]. Proportions ranged from 68 to 99% for this category. Six studies reported that 85% or more of clinicians often discuss PA with patients as part of their management plans, including providing advice, pertinent information or recommendations (i.e., specific exercise for fitness or disease prevention, awareness of national fitness objectives and amount of PA performed), in addition to relevant patient resources being available in the waiting room [24, 25, 30, 33–35]. In two studies there was less agreement on the importance of this, ranging from 68 to 77% of clinicians reporting that they frequently discussed fitness and/or exercise and provided PA advice or instruction in their initial chiropractic consultations [26, 36].

5. PA counselling

Three studies examined the frequency in which clinicians (n = 2188) counselled patients on PA. Proportions ranged from 50 to 81% for this category. One study reported that more than 80% of clinicians counselled patients at a frequency of once-to-several times per day, with respect to physical fitness and exercise promotion [27]. The two other studies reported that chiropractors typically provided counselling to 50% or more of their patients [29, 32].

## Discussion

This systematic review of surveys aimed to explore the attitudes and practice of chiropractors with respect to PA promotion within their clinics. To the best of our knowledge, this is the most up to date and comprehensive review of chiropractic PA promotion in practice.

### Main findings

We found a high proportion of chiropractors recognise the importance and are prepared to routinely discuss and/or counsel patients with respect to PA promotion. Our findings are consistent with a previous review, which reported that approximately 90% of chiropractors prescribed or advised on PA [14]; however this review was limited to three articles, whereas our review identified five times the number of surveys (n = 15). With respect to PA promotion, our review findings are in line with studies conducted with other AHPs [37], most notably physiotherapists and exercise physiologists [38, 39], which also report a strong recognition of the role of the health sector in promoting PA. While other AHPs generally agree that PA promotion is part of their role and they encourage PA engagement with their patients [38], the reasons for the high percentage of chiropractors promoting PA are not clear from our review. While chiropractors typically identify as spine and neuromusculoskeletal focused [40], they may also subscribe to and practice within a primary care and prevention framework, given the profession’s known (and traditional) focus on wellness [14]. Insights from the broader PA AHP literature suggest several factors can positively influence PA promotion, including regular PA engagement by the clinician [37], as well as their PA and sedentary behaviour guideline knowledge [37], skills and overall positive attitude toward the promotion of PA [41].

Our findings suggest that a high percentage of chiropractors gather information on PA participation from patients. For the most part, the specific nature of data collected in relation to PA is undefined. Thus, it is not clear whether chiropractors are conducting formal PA assessments as part of their routine information gathering. Other AHPs report often or always asking or screening their patients about PA [42]. This is often based on the patients past history, and their interest and ability to participate in PA [37]. Considering the comparable findings with other AHPs, chiropractors should be further encouraged to measure patient’s PA levels at each consultation. Given that PA is considered a vital sign i.e., an indicator of general physical condition, it can be tracked over time and compared with the most recently updated WHO PA and sedentary behaviour guidelines [43]. PA assessment procedures should ideally be simple, quick and user friendly in capturing information, particularly among those patients who could benefit from PA counselling [44]. This may include informal approaches by simply asking patients about their PA levels [45], to more formal assessments, such as utilising the reliable and valid General Practice Physical Activity Questionnaire to identify PA participation levels [46]. These approaches should be further explored and implemented within the chiropractic setting.

### Quality of studies

Our review is limited by the methodological weaknesses of the included studies, with all studies rated as moderate-to-high risk of bias. Therefore, proportions reported in our review should be interpreted with caution. All but one of the included studies were vulnerable due to ‘missing data’ for primary outcomes, which in relation to extracting data can further reduce confidence in the proportions reported in our review. Eighty-seven percent of included studies used surveys that were not formally validated, creating some uncertainty with respect to instrument quality and comparisons with prior established studies. Eighty-seven percent of studies did not achieve an ‘adequacy of response rate’, hence the low response rates in our included studies could lead to biased prevalence estimates. Eighty-seven percent of studies were not considered to include a representative population sample of a professional body or a defined geographical area, while 67% of studies conducted pilot testing, thus maximising greater survey user acceptability. A sensitivity analysis comprising only moderate risk of bias studies continued to show high a proportion of respondents, with discussing and providing PA ranging from 68 to 98%, while the importance of PA counselling and obtaining PA information from patients ranged from 64 to 99%. A universally accepted definition of discussing, counselling, and providing information with respect to PA, exercise and/or fitness promotion in chiropractic was not available in our studies. While this was anticipated, future standardisation of these and related PA outcome measurements would aid cross-study comparability.

## Strength and limitations

There are several strengths and limitations of this review, which should be acknowledged. We did not take trials or qualitative studies into account, although we plan to analyse known clinician barriers such as a perceived lack of time and lack of reimbursement, which has previously been reported among AHPs [47, 48]. Other limitations include the heterogeneity of the included studies, i.e., surveys performed in different settings using various sampling methods. Our method of grouping similar survey questions and subsequent responses required subjective judgments regarding sufficient similarity between different PA emerging categories. This may have resulted in misclassifications of PA concepts, i.e., practice categories identified and used in this review. This source of bias is acknowledged by illustrating the original survey questions asked in relation to PA promotion in Table 3. These allow readers to judge whether they agree with our PA practice categories or not. In addition, the presence of social desirability reporting bias within surveys (i.e., answering questions in a manner that will be viewed favorably by others) could have led to more favorable responses to questions from clinicians, thus potentially compromising (overestimating) the proportion of PA promotion reported in this review. Also, we did not locate the full surveys used in each study, thus in some cases the specific PA questions were not reportable. Notwithstanding, we provide an up-to-date systematic review examining chiropractor PA promotional and practice activities spanning three decades. Our review is based on 15 surveys, including almost 8,000 chiropractors, and is the most comprehensive systematic review on the topic to date. We pre-registered our protocol and utilised a comprehensive, updated search strategy, with no restrictions on language or publication date, thus reducing the likelihood of missing relevant surveys. We reported risk of bias on a domain-by-domain basis, and our review adhered to PRISMA guidelines, thus providing methodological rigour.

## Implications for research

The research survey output reporting PA promotional practice in chiropractic has increased slightly over the last 10 years, with a high proportion of respondents for PA promotion remaining constant over the last 30 years. This is consistent with the increased recognition of PA by the WHO and their more recent ‘whole systems approach’, in response to the complex physical inactivity public health challenge globally [7]. Despite this trend, the overall volume of literature with respect to chiropractic and PA promotion is both small and limited in study designs, compared to other AHPs [15]. Future evaluation may therefore consider interventional studies to inform effective PA integration into practice [15], as well as strategies to equip chiropractic clinicians to deliver the PA health advice. For instance, increasing clinician PA knowledge, confidence, and skills in conjunction with chiropractic associations or professional bodies, who could ‘scale up’ and support the PA promotional agenda by offering further relevant education and training. Notably, one study in our review identified a high percentage of chiropractors (i.e., 93%) believing it was either ‘very valuable’ or ‘valuable’ for clinicians to be educated or trained in specific subjects like exercise [23]. The need to educate and strengthen the training of AHPs in this field [49] is further highlighted by the WHO’s Global Action Plan on PA 2018–2030 [6]. Upskilling in the areas of PA assessment, advice and/or counselling, as well as behaviour change strategies and motivational interviewing, should be further explored [38].

Past reviews have explored HCPs own personal PA level, with some evidence suggesting higher participation in one’s own PA could translate into higher PA promotion in practice [41, 42, 50]. Studies within our review identified approximately 40% of chiropractors regularly participating in their own PA and/or exercise for fitness-related activities [22, 23, 25]. Therefore, any opportunity or potential benefits of increasing PA levels among chiropractors and subsequent role-modelling to patients should be considered [50]. This proposed research agenda should be feasible and compatible with clinical practice expectations [51]. It should also be encouraged globally within the chiropractic profession, given eligible studies in our review were drawn from only a small number of countries, limiting representation.

## Implications for practice

Although further enquiry is needed, chiropractors (like other AHPs) are well positioned to promote PA to their patients in practice. The nature of chiropractic practice is one of multiple patient-clinician interactions, where chiropractors build rapport and trust with their patients, creating a therapeutic alliance and the likelihood for important follow up consultations [52]. PA interventions delivered by health professionals in primary care are effective at increasing PA participation [10], hence multiple opportunities arise for chiropractors to impart and communicate the positive effects of PA on health-related quality of life by way of discussion, counselling or performing PA assessments. Realistically, chiropractic clinicians’ role as counsellors could readily extend into PA promotional activities, given they routinely counsel patients for musculoskeletal complaints.

## Conclusion

Our systematic review of cross-sectional surveys describes current attitudes and practices of chiropractors discussing, counselling, assessing, and providing information in relation to PA promotion. A high proportion of chiropractors surveyed are engaged in PA promotion, however methodological limitations within studies suggest caution be taken when interpreting results. Nevertheless, with the health benefits of PA well established, there is scope for chiropractors to be more involved in PA promotion in routine practice. Apart from the need for future unbiased surveys, forthcoming research initiatives should explore further PA education and training as well as capitalising on chiropractors’ own PA participation. This can further communicate the positive effects of PA on health-related quality of life by way of discussion, counselling or performing PA assessments.

## Supplementary Information

**Additional file 1**. Search strategy Medline, Mantis, AMED, EMBASE and Index to Chiropractic literature.

**Additional file 2**. PRISMA flowchart describing the process of study selection.

**Additional file 3**. Reasons for study exclusion.

## Data Availability

All data generated or analysed during this study are included in this published article.
