# Supplementary material for: Physical activity promotion in chiropractic: a systematic review of clinician-based surveys
Source: Chiropr Man Therap. 2022 Dec 13;30:55. doi: 10.1186/s12998-022-00467-9 (PMC9749165; doi:10.1186/s12998-022-00467-9)
Supplement: Supplementary file 1 — Additional file 1. Search strategy Medline, Mantis, AMED, EMBASE and Index to Chiropractic literature. [file 12998_2022_467_MOESM1_ESM.docx]

**Supplementary Table 1**

**Medline Search July 2020 PA**

|  |
| --- |
| 1. exp Exercise/ |
| 2. physical activit*.mp. |
| 3. exercis*.mp. |
| 4. Physical Fitness/ |
| 5. physical fitness.mp. |
| 6. movement.mp. |
| 7. motor activity/ |
| 8. 1 or 2 or 3 or 4 or 5 or 6 or 7 |
| 9. exp Health Promotion/ |
| 10. health promotion.mp. |
| 11. promot*.mp. |
| 12. advi#e.mp. |
| 13. counsel*.mp. |
| 14. wellness.mp. |
| 15. physical wellbeing.mp. |
| 16. 9 or 10 or 11 or 12 or 13 or 14 or 15 |
| 17. Chiropractic/ |
| 18. chiropract*.mp. |
| 19. practitioner.mp. |
| 20. physical therapist.mp. |
| 21. 17 or 18 or 19 or 20 |
| 22. 8 and 16 and 21 |
| 23. questionnaires/ |
| 24. survey*.mp. |
| 25. questionnaire*.mp. |

**Mantis August 2020 PA**

|  |  |  |  |
| --- | --- | --- | --- |
| 1. exp Exercise/ |  |  |  |
| 2. physical activit*.mp. |  |  |  |
| 3. exercis*.mp. |  |  |  |
| 4. Physical Fitness/ |  |  |  |
| 5. physical fitness.mp. |  |  |  |
| 6. movement.mp. |  |  |  |
| 7. motor activity/ |  |  |  |
| 8. 1 or 2 or 3 or 4 or 5 or 6 or 7 |  |  |  |
| 9. exp Health Promotion/ |  |  |  |
| 10. health promotion.mp. |  |  |  |
| 11. promot*.mp. |  |  |  |
| 12. advi#e.mp. |  |  |  |
| 13. counsel*.mp. |  |  |  |
| 14. wellness.mp. |  |  |  |
| 15. physical wellbeing.mp. |  |  |  |
| 16. 9 or 10 or 11 or 12 or 13 or 14 or 15 |  |  |  |
| 17. Chiropractic/ |  |  |  |
| 18. chiropract*.mp. |  |  |  |
| 19. practitioner.mp. |  |  |  |
| 20. physical therapist.mp. |  |  |  |
| 21. 17 or 18 or 19 or 20 |  |  |  |
| 22. 8 and 16 and 21 |  |  |  |
| 23. questionnaires/ |  |  |  |
| 24. survey*.mp. |  |  |  |
| 25. questionnaire*.mp. |  |  |  |

**Embase July 2020 PA**

|  |  |
| --- | --- |
| 1. exp Exercise/ |  |
| 2. physical activit*.mp. |  |
| 3. exercis*.mp. |  |
| 4. Physical Fitness/ |  |
| 5. physical fitness.mp. |  |
| 6. movement.mp. |  |
| 7. motor activity/ |  |
| 8. 1 or 2 or 3 or 4 or 5 or 6 or 7 |  |
| 9. exp Health Promotion/ |  |
| 10. health promotion.mp. |  |
| 11. promot*.mp. |  |
| 12. advi#e.mp. |  |
| 13. counsel*.mp. |  |
| 14. wellness.mp. |  |
| 15. physical wellbeing.mp. |  |
| 16. 9 or 10 or 11 or 12 or 13 or 14 or 15 |  |
| 17. Chiropractic/ |  |
| 18. chiropract*.mp. |  |
| 19. practitioner.mp. |  |
| 20. physical therapist.mp. |  |
| 21. 17 or 18 or 19 or 20 |  |
| 22. 8 and 16 and 21 |  |
| 23. questionnaires/ |  |
| 24. survey*.mp. |  |
| 25. questionnaire*.mp. |  |

**AMED August 2020**

|  |  |
| --- | --- |
| 1. exp Exercise/ |  |
| 2. physical activit*.mp. |  |
| 3. exercis*.mp. |  |
| 4. Physical Fitness/ |  |
| 5. physical fitness.mp. |  |
| 6. movement.mp. |  |
| 7. motor activity/ |  |
| 8. 1 or 2 or 3 or 4 or 5 or 6 or 7 |  |
| 9. exp Health Promotion/ |  |
| 10. health promotion.mp. |  |
| 11. promot*.mp. |  |
| 12. advi#e.mp. |  |
| 13. counsel*.mp. |  |
| 14. wellness.mp. |  |
| 15. physical wellbeing.mp. |  |
| 16. 9 or 10 or 11 or 12 or 13 or 14 or 15 |  |
| 17. Chiropractic/ |  |
| 18. chiropract*.mp. |  |
| 19. practitioner.mp. |  |
| 20. physical therapist.mp. |  |
| 21. 17 or 18 or 19 or 20 |  |
| 22. 8 and 16 and 21 |  |
| 23. questionnaires/ |  |
| 24. survey*.mp. |  |
| 25. questionnaire*.mp. |  |
| 26. cross-sectional stud*.mp. |  |
| 27. sampling stud*.mp. |  |
| 28. 23 or 24 or 25 or 26 or 27 |  |

**Index Chiropractic literature August 2020**

|  | [S1](https://www.chiroindex.org/?action=set&setId=5253072) | All Fields:Exercise |
| --- | --- | --- |
|  | [S3](https://www.chiroindex.org/?action=set&setId=5253108) | All Fields:Exercise* |
|  | [S4](https://www.chiroindex.org/?action=set&setId=5253117) | All Fields:physical activit* |
|  | [S5](https://www.chiroindex.org/?action=set&setId=5253125) | All Fields:Physical Fitness |
|  | [S6](https://www.chiroindex.org/?action=set&setId=5253133) | All Fields:exercis* |
|  | [S7](https://www.chiroindex.org/?action=set&setId=5253162) | All Fields:movement |
|  | [S8](https://www.chiroindex.org/?action=set&setId=5253169) | All Fields:Exercise OR All Fields:Exercise* OR All Fields:physical activit* OR All Fields:Physical Fitness OR All Fields:exercis* OR All Fields:movement |
|  | [S9](https://www.chiroindex.org/?action=set&setId=5253185) | All Fields:Health Promotion |
|  | [S10](https://www.chiroindex.org/?action=set&setId=5253192) | All Fields:promot* |
|  | [S11](https://www.chiroindex.org/?action=set&setId=5253198) | All Fields:advise |
|  | [S12](https://www.chiroindex.org/?action=set&setId=5253201) | All Fields:advice |
|  | [S13](https://www.chiroindex.org/?action=set&setId=5253208) | All Fields:counsel* |
|  | [S14](https://www.chiroindex.org/?action=set&setId=5253213) | All Fields:wellness |
|  | [S15](https://www.chiroindex.org/?action=set&setId=5253220) | All Fields:Health Promotion OR All Fields:promot* OR All Fields:advise OR All Fields:advice OR All Fields:counsel* OR All Fields:wellness |
|  | [S17](https://www.chiroindex.org/?action=set&setId=5253267) | All Fields:Chiropractic* |
|  | [S18](https://www.chiroindex.org/?action=set&setId=5253272) | All Fields:chiropract* |
|  | [S19](https://www.chiroindex.org/?action=set&setId=5253282) | All Fields:practitioner* |
|  | [S20](https://www.chiroindex.org/?action=set&setId=5253284) | All Fields:Chiropractic* OR All Fields:chiropract* OR All Fields:practitioner* |
|  | [S21](https://www.chiroindex.org/?action=set&setId=5253288) | All Fields:Exercise OR All Fields:Exercise* OR All Fields:physical activit* OR All Fields:Physical Fitness OR All Fields:exercis* OR All Fields:movement AND All Fields:Health Promotion OR All Fields:promot* OR All Fields:advise OR All Fields:advice OR All Fields:counsel* OR All Fields:wellness AND All Fields:Chiropractic* OR All Fields:chiropract* OR All Fields:practitioner* |
|  | [S22](https://www.chiroindex.org/?action=set&setId=5253289) | All Fields:questionnaire* |
|  | [S23](https://www.chiroindex.org/?action=set&setId=5253290) | All Fields:survey* |
|  | [S25](https://www.chiroindex.org/?action=set&setId=5253293) | All Fields:surveys and questionnaires |
|  | [S26](https://www.chiroindex.org/?action=set&setId=5253294) | All Fields:Cross-Sectional Studies* |
|  | [S27](https://www.chiroindex.org/?action=set&setId=5253297) | All Fields:Sampling Studies |
|  | [S28](https://www.chiroindex.org/?action=set&setId=5253300) | All Fields:questionnaire* OR All Fields:survey* OR All Fields:surveys and questionnaires OR All Fields:Cross-Sectional Studies* OR All Fields:Sampling Studies |
|  | [S29](https://www.chiroindex.org/?action=set&setId=5253301) | All Fields:Exercise OR All Fields:Exercise* OR All Fields:physical activit* OR All Fields:Physical Fitness OR All Fields:exercis* OR All Fields:movement AND All Fields:Health Promotion OR All Fields:promot* OR All Fields:advise OR All Fields:advice OR All Fields:counsel* OR All Fields:wellness AND All Fields:Chiropractic* OR All Fields:chiropract* OR All Fields:practitioner* AND All Fields:questionnaire* OR All Fields:survey* OR All Fields:surveys and questionnaires OR All Fields:Cross-Sectional Studies* OR All Fields:Sampling Studies |
