# Supplementary material for: Physical activity promotion in chiropractic: a systematic review of clinician-based surveys
Source: Chiropr Man Therap. 2022 Dec 13;30:55. doi: 10.1186/s12998-022-00467-9 (PMC9749165; doi:10.1186/s12998-022-00467-9)
Supplement: Supplementary file 2 — Additional file 2. PRISMA flowchart describing the process of study selection. [file 12998_2022_467_MOESM2_ESM.doc]

**Screening**

**Included**

**Eligibility**

**Identification**

Records identified through 5 database searches

Medline (217)

Mantis (73)

Embase (277)

AMED (36)

Index to chiropractic literature (57)
(n=661)

Records after duplicates removed
(n=408)

Full text articles screened for eligibility
(n=45)

Studies included in qualitative synthesis
(n=15)

hk

Studies included in quantitative synthesis (meta-analysis)
(n=15)

FiFikar,

Title and abstract screened

(n=408)

Additional records identified through other sources
Previously identified (n=4)

Citation searches (n=2)

Records excluded by title and abstract
(n =363)

Full-text articles excluded (n=30)

Reasons:
Questionnaire, no PA (n=5)

Survey, no PA (n=11)

Review (n=1)

Practice based research network, no PA (n=1)

Secondary data analysis (n=3)

Delphi study design (n=1)

Clinical trial design (n=1)

Case study design (n=3)

Mixed methods design, no PA (n=1)

Qualitative design (n=3)
