# Supplementary material for: Physical activity promotion in chiropractic: a systematic review of clinician-based surveys
Source: Chiropr Man Therap. 2022 Dec 13;30:55. doi: 10.1186/s12998-022-00467-9 (PMC9749165; doi:10.1186/s12998-022-00467-9)
Supplement: Supplementary file 3 — Additional file 3. Reasons for study exclusion. [file 12998_2022_467_MOESM3_ESM.docx]

**Supplementary Table 2**

| **Study title** | **Design** | **Wrong populations and/or outcomes** | **Wrong study design** |
| --- | --- | --- | --- |
| Ailliet L, Rubinstein SM, de Vet HC. Characteristics of chiropractors and their patients in Belgium. Journal of manipulative and physiological therapeutics. 2010 Oct 1;33(8):618-25. | Questionnaire | ● |  |
| Gleberzon BJ. Developing a community-based educational program for older persons. The Journal of the Canadian Chiropractic Association. 2001 Mar;45(1):18. | Narrative review |  | ● |
| DuMonthier WN, Haneline MT, Smith M. Survey of health attitudes and behaviors of a chiropractic college population. Journal of manipulative and physiological therapeutics. 2009 Jul 1;32(6):477-84. | Survey | ● |  |
| Evans Jr MW, Page G, Ndetan H, Martinez D, Brandon P, Daniel D, Walker C. Are Patients Receiving Health Promotion Advice in the Chiropractic Teaching Clinic Setting? An Impact Assessment of a Brief Intervention to Increase Advising Rates and Goal Setting. Journal of Chiropractic Education. 2011 Oct;25(2):132-41. | Survey | ● |  |
| Evans MW, Hawk C, Ndetan H, Rupert R. Patient characteristics, screening use, and health education advice in a chiropractic practice-based research network. Top Integrative Health Care. 2012;3(1):1-4. | Cross-sectional Practice-Based Research Network | ● |  |
| Fernandez M, Moore C, Eklund A, Swain M, de Luca K, Sibbritt D, Adams J, Peng W. The prevalence and determinants of physical activity promotion by Australian chiropractors: A cross sectional study. Complementary therapies in medicine. 2019 Aug 1;45:172-8. | Secondary data analysis |  | ● |
| Howitt S, Ethridge E, Nelson E, Gotuaco M, Demello L. Exercise prescription: perceptions and physical activity habits in chiropractic students at CMCC. The Journal of the Canadian Chiropractic Association. 2016 Dec;60(4):286. | Survey | ● |  |
| Jamison JR. Stress management: An exploratory study of chiropractic patients. Journal of manipulative and physiological therapeutics. 2000 Jan 1;23(1):32-6. | Qualitative |  | ● |
| Jamison JR. Wellness from the perspective of Australian chiropractic patients. Chiropractic Journal of Australia. 2007 Mar;37(1):11-4. | Survey | ● |  |
| Jamison JR. Prescribing wellness: a case study exploring the use of health information brochures. Journal of manipulative and physiological therapeutics. 2004 May 1;27(4):262-6. | Questionnaire | ● |  |
| Jamison JR, Geraghty B, Keating G, Livingstone K. Osteoporosis screening and prevention in the chiropractic clinic. Journal of Manipulative and Physiological Therapeutics. 1988 Oct 1;11(5):390-5. | Survey | ● |  |
| Jamison J. Stress: the chiropractic patients' self-perceptions. Journal of manipulative and physiological therapeutics. 1999 Jul 1;22(6):395-8. | Questionnaire | ● |  |
| Jamison JR. Fostering critical appraisal skills as a prelude to clinical practice. Chiropractic Journal of Australia. 2005 Sep;35(3):107-11. | Case study |  | ● |
| Jamison JR. Fostering critical thinking skills: a strategy for enhancing evidence-based wellness care. Chiropractic & Osteopathy. 2005 Dec;13(1):1-9. | Case study |  | ● |
| Jamison JR. Wellness: Defining the Way Ahead for Chiropractic in Australia? Chiropractic Journal of Australia. 2007 Mar;37(1):2-6. | Delphi study |  | ● |
| Mistry RA, Bacon CJ, Moran RW. Attitudes and self-reported practices of New Zealand osteopaths to exercise consultation. International Journal of Osteopathic Medicine. 2018 Jun 1;28:48-55. | Questionnaire | ● |  |
| Ndetan H, Evans Jr MW, Felini M, Bae S, Rupert R, Singh KP. Chiropractic and medical use of health promotion in the management of arthritis: analysis of the 2006 National Health Interview Survey. Journal of manipulative and physiological therapeutics. 2010 Jul 1;33(6):419-24. | Secondary data analysis |  | ● |
| Ndetan HT, Bae S, Evans Jr MW, Rupert RL, Singh KP. Characterization of health status and modifiable risk behavior among United States adults using chiropractic care as compared with general medical care. Journal of manipulative and physiological therapeutics. 2009 Jul 1;32(6):414-22. | Secondary data analysis |  | ● |
| Nelson L, Pollard H, Ames R, Jarosz B, Garbutt P, Da Costa C. A descriptive study of sports chiropractors with an International Chiropractic Sport Science Practitioner qualification: a cross-sectional survey. Chiropractic & Manual Therapies. 2021 Dec;29(1):1-6. | Survey | ● |  |
| Newell D, MChiro RB. Increasing compliance toward home exercise in chiropractic patients using SMS texting: A pilot study. Clinical Chiropractic. 2012 Dec 1;15(3-4):107-11. | Pilot clinical trial |  | ● |
| Puhl AA, Reinhart CJ, Injeyan HS. Diagnostic and treatment methods used by chiropractors: A random sample survey of Canada’s English-speaking provinces. The Journal of the Canadian Chiropractic Association. 2015 Sep;59(3):279. | Questionnaire | ● |  |
| Rupert RL, Manello D, Sandefur R. Maintenance care: health promotion services administered to US chiropractic patients aged 65 and older, part II. Journal of manipulative and physiological therapeutics. 2000 Jan 1;23(1):10-9. | Survey | ● |  |
| Short CE, Hayman M, Rebar AL, Gunn KM, De Cocker K, Duncan MJ, Turnbull D, Dollman J, van Uffelen JG, Vandelanotte C. Physical activity recommendations from general practitioners in Australia. Results from a national survey. Australian and New Zealand journal of public health. 2016 Feb;40(1):83-90. | Survey | ● |  |
| Stainsby BE, Porr JT, Kim P, Collinge AM, Hunter JC. A survey of wellness management strategies used by Canadian Doctor of Chiropractic. Journal of manipulative and physiological therapeutics. 2011 Jul 1;34(6):388-93. | Qualitative |  | ● |
| Weinert D, McDermott AM. Academic, and clinical design to promote utilization of active care procedures. JOURNAL OF SPORTS CHIROPRACTIC & REHABILITATION. 2000 Mar 1;14(1):21-3. | Case study |  | ● |
| Lee AD, Szabo K, McDowell K, Granger S. Opinions of sports clinical practice chiropractors, with sports specialty training and those without, about chiropractic research priorities in sports health care: a centering resonance analysis. The Journal of the Canadian Chiropractic Association. 2016 Dec;60(4):342. | Qualitative |  | ● |
| Hawk C, Dusio ME. Chiropractors’ attitudes toward training in prevention: results of a survey of 492 U.S. chiropractors. J Manipulative Physiol Ther. 1995;18:135-140 | Survey | ● |  |
| Jamison JR, Rupert RL. Maintenance care: towards a global description. The Journal of the Canadian Chiropractic Association. 2001 Jun;45(2):100. | Survey | ● |  |
| Mootz RD, Cherkin DC, Odegard CE, Eisenberg DM, Barassi JP, Deyo RA. Characteristics of chiropractic practitioners, patients, and encounters in Massachusetts and Arizona. Journal of manipulative and physiological therapeutics. 2005 Nov 1;28(9):645-53. | Mixed methods |  | ● |
| Jacobson BH, Gemmell HA: A survey of chiropractors in Oklahoma. J Chiropr Educ. 1999, 13: 137-142. | Survey | ● |  |
